# A retrospective study on molecular epidemiology trends of carbapenem resistant *Enterobacteriaceae* in a teaching hospital in Malaysia

**DOI:** 10.7717/peerj.12830

**Published:** 2022-02-22

**Authors:** Zhi Xian Kong, Rina N. Karunakaran, Kartini Abdul Jabar, Sasheela Ponnampalavanar, Chun Wie Chong, Cindy Shuan Ju Teh

**Affiliations:** 1 Department of Medical Microbiology, University of Malaya, Kuala Lumpur, Wilayah Persekutuan Kuala Lumpur, Malaysia; 2 Department of Medicine, University of Malaya, Kuala Lumpur, Wilayah Persekutuan Kuala Lumpur, Malaysia; 3 School of Pharmacy, Monash University Malaysia, Bandar Sunway, Selangor, Malaysia

**Keywords:** Carbapenem resistant *Enterobacteriaceae*, Carbapenemase, Pulse-field gel electrophoresis, Porins, Epidemiology

## Abstract

**Background:**

Carbapenem resistant *Enterobacteriaceae* (CRE) has rapidly disseminated worldwide and has become a global threat to the healthcare system due to its resistance towards “last line” antibiotics. This study aimed to investigate the prevalence of CRE and the resistance mechanism as well as the risk factors associated with in-hospital mortality.

**Methods:**

A total of 168 CRE strains isolated from a tertiary teaching hospital from 2014–2015 were included in this study. The presence of carbapenemase genes and minimum inhibitory concentration of imipenem, meropenem and colistin were investigated. All carbapenem-resistant *Klebsiella pneumoniae* (*K. pneumoniae*) strains were characterised by PFGE. The risk factors of patients infected by CRE associated with in-hospital mortality were determined statistically.

**Results:**

The predominant CRE species isolated was *K. pneumoniae*. The carbapenemases detected were *bla*OXA-48, *bla*OXA-232, *bla*VIM and *bla*NDM of which *bla*OXA-48 was the predominant carbapenemase detected among 168 CRE strains. A total of 40 CRE strains harboured two different carbapenemase genes. A total of seven clusters and 48 pulsotypes were identified among 140 CRKp strains. A predominant pulsotype responsible for the transmission from 2014 to 2015 was identified. Univariate statistical analysis identified that the period between CRE isolation and start of appropriate therapy of more than 3 days was statistically associated with in-hospital mortality.

## Introduction

Carbapenem resistance is a recognised worldwide threat by the World Health Organisation (WHO). One of the major causes for the emergence of carbapenem resistant pathogens is the overuse of carbapenems in the management of infections caused by extended-spectrum beta-lactamases (ESBLs)-expressing *Enterobacteriaceae* (Nordmann, Dortet & Poirel, 2012; Slama, 2008; Rahal, 2008). Indeed, given that carbapenem is recommended for the treatment of ESBL infections (Paterson et al., 2004), a steady rise in carbapenem resistance had been recorded in Malaysia with the increasing use of the antibiotics (Institute for Medical Research, 2020).

Carbapenem-resistant *Klebsiella pneumoniae* (CRKp) has persisted and has been gradually rising since its first discovery. For instance, in southeastern United States, a five-fold increase in CRKp infections were recorded from 2008 to 2012 (Thaden et al., 2014). Although carbapenem resistance data is limited, particularly in Southeast Asia, a meta-analysis revealed high estimated carbapenem resistance among *Klebsiella* spp. in Indonesia, the Philipines, Thailand and Vietnam and among *E. coli* in Indonesia and Myanmar (Malchione et al., 2019). Among the recorded carbapenem resistant *Enterobacteriaceae* (CRE), *K. pneumoniae* is the most prevalent species in Malaysia (Institute for Medical Research, 2020; Zaidah et al., 2017).

The widespread distribution of CRKp over different geographical regions is commonly associated with high-risk clones and the acquisition of carbapenemase genes encoded in mobile genetic elements (David et al., 2019; Pitout, Nordmann & Poirel, 2015). In a parallel survey by the European survey of carbapenemase-producing *Enterobacteriaceae* (EuSCAPE), a 1.3% increase in population-weighted mean percentage in carbapenem-resistant *K. pneumoniae* and *Escherichia coli* (*E. coli*) was recorded in EU member states from 2011–2014 (Xu et al., 2015; Thaden et al., 2014; European Centre for Disease Prevention and Control, 2020). In Malaysia, the reported carbapenemases that produced by *Enterobacteriaceae* are *bla*NDM, *bla*IMP, *bla*OXA-48, *bla*OXA-181 and *bla*KPC (Hamzan et al., 2015; Wang et al., 2016; Lau et al., 2020).

CRE is commonly associated with nosocomial infection. A previous study had identified several factors associated with CRE acquisition such as previous overseas hospitalisation, ICU admission and comorbidities (cardiovascular disease and hematology condition) (Ling et al., 2015). Furthermore, studies had also revealed that usage of mechanical ventilation, presence of central venous catheter and receiving immunosuppressants were associated with mortality (Low et al., 2017; Wang et al., 2016). Understanding the transmission of CRE as well as their resistance mechanism is essential to inform the infection control strategy for containing the spread of CRE. In this study, we investigated the epidemiology of CRE (from 2014 to 2015) in a teaching hospital in Klang Valley, Malaysia. The risk factors associated with mortality among the patients infected or colonised with CRE was also examined.

## Materials and Methods

### Ethics statement

The medical ethics approval for clinical strains collection and clinical/patients’ data access was obtained from University of Malaya Medical Centre (UMMC) Medical Research Ethics Committee in 2015 (MECID.NO: 20154-1249). In this retrospective study, the informed consent from patient is not required.

### Hospital setting and bacterial collection

Strain collection was conducted in UMMC, a tertiary teaching and public hospital with 1,617 beds located in Klang Valley, Malaysia. The *Enterobacteriaecae* clinical cultures that had been identified as carbapenem resistant in the Medical Microbiology Diagnostic Laboratory (MMDL) of UMMC were retrospectively collected and included in the study. All the included strains were the first CRE isolated and stocked from the patients’ samples between 1^st^ of January of 2014 to 31^st^ of December of 2015. When different sample types received on the same day from a patient had grown CRE, only the first strain per patient which was stocked was included.

### Detection of carbapenemase genes among CRE strains

Crude DNA from the CRE strains was prepared using direct boiling method as mentioned by Lim, Teh & Thong (2013). The presence of carbapenemase genes such as *bla*KPC, *bla*NDM, *bla*VIM, *bla*IMP, *bla*OXA among the CRE strains were identified by polymerase chain reaction (PCR) as previously described by Naas et al. (2008), Nordmann, Naas & Poirel (2011), Poirel et al. (2011), Poirel et al. (2011), and Poirel et al. (2004) respectively. The amplicons were analysed on 1.0% agarose gel.

### Evaluation of antibiotic susceptibility

The MIC value of imipenem, meropenem and colistin among the CRE strains were determined through broth microdilution method according to the Clinical and Laboratory Standard Institute (CLSI) guidelines (Clinical and Laboratory Standards Institute (CLSI), 2021). The MIC breakpoints from the CLSI guidelines was used for imipenem and meropenem (Clinical and Laboratory Standards Institute (CLSI), 2021) while European Committee on Antimicrobial Susceptibility Testing (EUCAST) was used to interpret for the susceptibility of colistin (The European Committee on Antimicrobial Susceptibility Testing (EUCAST), 2021). According to CLSI guidelines, the MIC breakpoints of imipenem and meropenem for susceptible, intermediate, and resistant are ≤1, 2, ≥4 µg/mL respectively. Whilst, according to EUCAST, the MIC breakpoints of colistin for susceptible and resistant are ≤2 and >2 µg/mL respectively. The antibiotic susceptibility of ertapenem was obtained from Medical Microbiology Diagnostic Laboratory (MMDL) of UMMC where the disc diffusion test was carried out. The results were re-interpreted according to CLSI guidelines year of 2021 (Clinical and Laboratory Standards Institute (CLSI), 2021).

### Clinical data collection

The clinical details at the time of the CRE isolation were obtained from the patient’s case notes. These details are clearly characterised: (1) A CRE colonisation is defined as the isolation of the microorganism from any non-sterile body sites (usually rectum or perianal area, and respiratory tract/oral cavity, vagina, skin and urine) in the absence of clinical findings of infection (Girmenia et al., 2015). (2) Hospital-acquired infection is defined as infection with positive culture obtained from patients already hospitalised for 48 h or longer (Friedman et al., 2002). Healthcare-associated infection was defined as infection with positive culture obtained at admission or within 48 h or from someone with history of previous hospitalisation or medical procedures (Friedman et al., 2002). (3) Empirical antibiotics is defined as antibiotic that is given to the patients with sign and symptoms of infection before the identification of organisms and susceptibility data were available. (4) Targeted antibiotic is defined as antibiotic that is given to the patients after the culture and susceptibility data is available. (5) Invasive device is defined as a medical device that is introduced into the body, either through a break in the skin or body orifice. This includes central venous line, peripheral line, peripherally inserted central catheter, nasogastric tube, catheter bladder drainage, internal jugular catheter and external ventricular drain. (6) An invasive procedure is defined as one where access to the body is gained *via* an incision, percutaneous puncture, where instrumentation is used in addition to the puncture needle, or instrumentation *via* a natural orifice. Invasive procedures include, but are not limited to, endoscopes, catheters, scalpels, scissors, devices, and tubes (Cousins, Blencowe & Blazeby, 2019). (7) Underlying disorder is defined as an acute or chronic comorbidity or condition not related to sepsis that alters short- and/or long-term survival of infectious diseases (Dhainaut et al., 2005). (8) Length of hospitalisation is defined as length of inpatient care, which calculated from the day of admission to the day of discharge. (9) Appropriate therapy is defined as the use of antimicrobial agents with *in-vitro* activity against the etiologic pathogens (American Thoracic Society, 2005).

### Molecular detection of porin genes in *Klebsiella* spp.

The presence of porin associated genes such as *ompK35*, *ompK36* and *ompK37* among the *Klebisella* spp. were determined by PCR as previously described by Kaczmarek et al. (2006). The PCR product was analysed on 1.0% agarose gel.

### Clonal relatedness of carbapenem resistant *Klebsiella pneumoniae*

The clonal relationship between CRKp strains were investigated using pulse-field gel electrophoresis (PFGE). Plug consisted of extracted whole genomic DNA were digested by *Xba*I restriction enzyme as previously described by Low et al. (2017). The banding patterns produced were analysed using BioNumerics software (AppliedMaths, Philadelphia, PA, USA). Unweighted Pair Group Method with Arithmetic (UPGMA) were utilised for the cluster analysis with Dice correlation coefficient and 1.5% of tolerance and 1.0% optimisation. The clustering analysis was performed based on the similarity value of ≥80%.

### Statistical analysis

The risk factors associated with mortality were deduced statistically using statistical software SPSS 2.0. All continuous variables were expressed as mean ± standard deviation. Nominal variables were evaluated by Fishers’ exact test or chi-square as appropriate while continuous variables were evaluated by student t test or Mann Whitney U test based on the data normality. The analysis was deemed as statistically significant if the *P* value were less than 0.05.

## Results

### Identification of CRE samples in patients’ samples

A total of 34 (from 34 patients) and 134 (from 134 patients) CRE strains were retrospectively collected from 2014 and 2015, respectively. *K. pneumoniae* was the dominant species isolated in both 2014 and 2015, contributed to 73.5% (*n* = 25) and 85.8% (*n* = 115) of the total CRE strains in 2014 and 2015, respectively (Table 1). In 2014, a small number of *Enterobacter cloacae* (*n* = 4, 11.8%), *Escherichia coli* (*n* = 1, 2.9%), *Citrobacter freundii* (*n* = 2, 5.9%) and *Klebsiella oxytoca* (*n* = 2, 5.9%) were collected. In 2015, the non-*Klebsiella* species isolated were *Escherichia coli* (*n* = 11, 8.3%), *Citrobacter freundii* (*n* = 2, 1.5%), *Enterobacter cloacae* (*n* = 2, 1.5%), *Serratia marcescens* (*n* = 2, 1.5%), *Citrobacter koseri* (*n* = 1, 0.7%) and *Enterobacter aerogenes* (*n* = 1, 0.7%). Blood (*n* = 35, 20.8%), urine (*n* = 29, 17.3%) and tracheal secretion (*n* = 22, 13.1%) were the common specimens that included in this study (Table 1).

**Table 1:** Comparison between CRE samples type, identified CRE species and carbapenemase genes detected in 2014 and 2015.

	2014 (*n* =)	2015 (*n* =)
**Patient samples**		
Blood	5	30
Urine	8	21
Tracheal secretion	5	17
Sputum	2	13
Bronchoalveolar fluid	3	4
Pleural fluid	1	0
Pus	1	0
Nasopharyngeal secretion	1	2
Respiratory secretion	0	1
Swab	0	5
Peritoneal fluid swab	1	3
Peritoneal slough swab	1	0
Bone swab	0	2
Backbone suction swab	0	2
Cerebrospinal fluid swab	0	1
Bile swab	0	1
Abdomen fluid swab	0	1
Cyst swab	0	1
Fluid swab	0	1
Sacral swab	1	2
Groin swab	1	1
Stoma swab	1	0
Tissue swab	0	6
Wound swab	2	7
Breast swab	0	1
Pus swab	0	5
Ear swab	0	1
Rectal swab	1	1
Tip swab	0	0
Pigtail aspirate swab	0	1
Chest tube swab	0	1
Catheter swab	0	1
Latex A fluid swab	0	1
Eye swab	0	1
**Identified species**		
*Klebsiella pneumoniae*	25	115
*Escherichia coli*	1	11
*Enterobacter cloacae*	4	2
*Serratia marcescens*	0	2
*Citrobacter freundii*	2	2
*Citrobacter koseri*	0	1
*Klebsiella oxytoca*	2	0
*Enterobacter aerogenes*	0	1
**Carbapenemase genes**		
*bla*NDM	19	53
*bla*OXA-48	11	103
*bla*OXA-232	0	3
*bla*VIM	0	1
*bla*KPC	0	0
*bla*IMP	0	0
No targeted genes detected	5	13
**Loss of porin ( *Klebsiella spp*.)**		
*ompK35*	3	3
*ompK36*	3	5
*ompK37*	6	15

### Demographic and clinical characteristic of patients

A total of 168 CRE from 168 patients was included in the study; however, 27 patients were excluded from statistical analysis as the data was incomplete in the system. For the 141 sets of patient data extracted from the system, no significant differences in age, races and gender were detected between survivors and non-survivors (Table 2). The mean age of the patients was 54.93 ± 21.42. Chinese were the predominant race (*n* = 52, 36.9%) followed by Malays (*n* = 47, 33.3%), Indians (*n* = 33, 23.4%) and others (*n* = 9, 6.4%). There were 91 male and 50 female patients. The average length of hospitalisation of patients was more than a month which was 41.15 ± 30.74 days. The survivors recorded a longer duration of hospitalisation. From the 141 CRE isolated, 53.2% (*n* = 75) were associated with infection while 46.8% (*n* = 66) were colonisers. The all-cause mortality among patients with CRE isolation (infection/colonisation) was 39.7% (*n* = 56). Additionally, the duration of CRE isolation and appropriate therapy of more than 3 days was statistically associated with higher mortality.

**Table 2:** The demographic and clinical data of patients associated with in-hospital all-cause mortality.

Variables	Survivors (*n* = 85)	Non-survivors (56)	*P* value
**Age (years)**	54.21 ± 21.99	56.02 ± 20.67	0.610^b^
**Ethnicity**			
**Malay**	28	19	0.903
**Chinese**	32	20	0.816
**Indian**	19	14	0.716
**Other**	6	3	1.000^a^
**Gender**			
**Male**	60	31	0.064
**Female**	25	25	0.064
**Length of hospitalisation (days)**	44.98 ± 31.75	35.34 ± 28.45	0.035^b^
**Underlying disorder**			
**Diabetes mellitus**	25	21	0.316
**Hypertension**	32	28	0.147
**End-stage renal disease/kidney disease**	11	21	0.000
**Malignancy**	13	12	0.501
**Respiratory disease**	3	3	1.000^a^
**Brain disease**	7	4	1.000^a^
**Endocrine/metabolite disease**	11	4	0.275
**Urinary/bladder disease**	4	0	0.152^a^
**Gastrointestinal disease**	8	4	0.763^a^
**Bone/joint disease**	3	3	0.682^a^
**Skin disease**	9	3	0.363^a^
**Polytrauma**	5	2	0.703^a^
**Liver/pancreas disease**	8	5	0.923
**Tissue disease**	3	0	0.277^a^
**Blood disorder**	4	7	0.114^a^
**Neurological disease**	4	6	0.195^a^
**Heart disease**	5	5	0.518^a^
**Pleural effusion**	2	1	0.518^a^
**Bowel dislocation**	1	0	1.000^a^
**Rectal prolapse**	1	0	1.000^a^
**Infection**	34	28	0.242
**Prostate disease**	1	1	1.000^a^
**Immune disease**	0	1	0.397^a^
**Parotitis**	0	1	0.397^a^
**None**	5	0	0.157^a^
**Invasive device/procedure**	81 (95.3%)	56 (100%)	0.152^a^
**ICU stay**	50 (58.8%)	37 (66.1%)	0.479^a^
**Colonisation/infection**			
**Colonisation**	52 (61.2%)	14 (25.0%)	0.000
**Infection**	33 (38.8%)	42 (75.0%)	0.000
**Period between isolation and appropriate therapy for infection cases (*n* = 75)**			0.048
**≤3**	15	10	
**>3**	18	32	
**Carbapenemase**			
**OXA-48**	38 (44.7%)	24 (42.9%)	0.829
**NDM**	16 (18.8%)	11 (19.6%)	0.904
**OXA-232**	1 (1.2%)	1 (1.8%)	1.000^a^
**OXA-48+NDM**	18 (21.2%)	16 (28.6%)	0.315
**No genes**	12 (14.1%)	4 (7.1%)	0.201

**Note:**

*P* were obtained using chi-square test unless stated otherwise. *P*-values^a^ was obtained using Fishers’ exact test. *P*-values^b^ was obtained using Mann Whitney U test.

### Distribution of carbapenemase genes among CRE strains

The dominant carbapenemase gene detected among the 2014 strains was *bla*NDM (*n* = 19, 55.9%) followed by *bla*OXA-48 (*n* = 11, 32.4%). In 2015, *bla*OXA-48 (*n* = 103, 76.9%) was the predominant carbapenemase gene among the CRE strains, followed by *bla*NDM (*n* = 53, 39.6%), *bla*OXA-232 (*n* = 3, 2.2%) and *bla*VIM (*n* = 1, 0.7%) (Table 1). Both *bla*IMP and *bla*KPC were not detected and 18 strains did not harbour any targeted carbapenemase genes. In 2014, 28 (82.4%) strains harboured single carbapenemase gene and one (2.9%) strain harboured dual carbapenemase genes (*bla*OXA-48+*bla*NDM). While in 2015, 82 (61.2%) strains harboured single carbapenemase gene and 39 (29.1%) strains harboured dual carbapenemase genes (1 strain with *bla*OXA-232+*bla*VIM, 38 strains with *bla*OXA-48+*bla*NDM).

### Differential sensitivity to carbapenem/colistin among the CRE clinical strains

A high prevalent of resistant to imipenem and meropenem was detected (Table 3). Based on the CLSI guidelines, majority of the strains were either intermediate or resistant to imipenem (82.2%) and meropenem (74.4%). A total of 14 (8.3%) CRE strains exhibited MIC ≤ 1 for both imipenem and meropenem but were mono-resistant/intermediate to ertapenem. Among these 14 strains, 10 strains (71.4%) were *K. pneumoniae*, 2 strains (14.3%) were *E. coli* and 1 strain (7.1%) each was *Enterobacter cloacae* and *Citrobacter freundii* respectively. In addition, 11 (6.5%) strains were colistin-resistant. Among these 11 strains, 10 strains (90.9%) were *K. pneumoniae* and 1 strain (9.1%) was *Serratia marcescens* (Table 3).

**Table 3:** Sensitivity to imipenem, meropenem and colistin among CRE strains.

	Sensitive (≤1 µg/mL) (*n* =)	Intermediate (2 µg/mL) (*n* =)	Resistant (≥4 µg/mL) (*n* =)
**Imipenem**			
*Klebsiella pneumoniae*	25	19	96
*Escherichia coli*	2	5	5
*Enterobacter cloacae*	2	2	2
*Serratia marcescens*	0	0	2
*Citrobacter freundii*	1	1	2
*Escherichia coli*	0	0	1
*Citrobacter koseri*	0	0	0
*Klebsiella oxytoca*	0	1	1
*Enterobacter aerogenes*	0	0	1
**Meropenem**			
*Klebsiella pneumoniae*	30	18	92
*Escherichia coli*	7	1	4
*Enterobacter cloacae*	2	1	3
*Serratia marcescens*	1	0	1
*Citrobacter freundii*	2	1	1
*Citrobacter koseri*	0	0	1
*Klebsiella oxytoca*	1	0	1
*Enterobacter aerogenes*	0	1	0
	**Sensitive (≤2 µg/mL) (*n* =)**		**Resistant (>2 µg/mL) (*n* =)**
**Colistin**			
*Klebsiella pneumoniae*	130		10
*Escherichia coli*	12		0
*Enterobacter cloacae*	6		0
*Serratia marcescens*	1		1
*Citrobacter freundii*	4		0
*Citrobacter koseri*	1		0
*Klebsiella oxytoca*	2		0
*Enterobacter aerogenes*	1		0

### Detection of porin genes associated within *Klebsiella spp*.

Of the 27 *Klebsiella spp*. isolated in 2014, nine strains (33%) loss either 1 or 2 porin(s). The number of strains with at least 1 porin loss reduced in 2015 (*n* = 23; 20%), but 2 strains loss all 3 porins (Table 1).

### Molecular characterization of CRKp by PFGE

**Figure 1:**
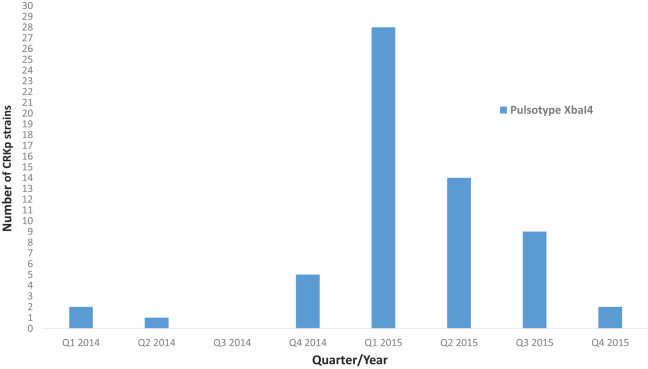
The occurrence of pulsotype *Xba*I4 among CRKp strains from 2014 to 2015. Sporadic isolation of pulsotype *Xba*I4 was observed in the first two quarters of 2014. However, there was a steep increment in the isolation of this pulsotype from Q4 2014 to Q1 2015. A gradual decrement in the isolation of pulsotype *Xba*I4 has been observed from Q2 of 2015.

Based on dendrogram (S1–S3), the 140 CRKp strains were separated into seven major clusters (Cluster I, II, III, IV, V, VI, VII). More than half of the CRKp strains (*n* = 81, 57.6%) were clustered into Cluster I (11 pulsotypes) followed by Cluster IV (*n* = 8, 5.7%) and V (*n* = 7, 5.0%). A total of 48 pulsotypes (*Xba*I1–*Xba*I48) have been generated from the PFGE analysis. Among the detected pulsotypes, pulsotype *Xba*I4. which belonged to Cluster I is the predominant pulsotype that consists of 62 strains (44.3%) which is 100% clonally similar to each other. This pulsotype has been sporadically isolated in January (*n* = 1), February (*n* = 1) and April (*n* = 1) of 2014. However, a steep increment of this pulsotype was observed since December 2014 (*n* = 5) until the first quarter of 2015 (*n* = 28). The isolation of this pulsotype was later decreased from 2^nd^ quarter of 2015 (Fig. 1).

## Discussion

*K. pneumoniae* is a human commensal that colonises the mucosal surfaces such as gastrointestinal tract and respiratory tract (Podschun & Ullmann, 1998). Gastrointestinal tract has been reported as the important reservoir for the transmission and infection of resistant pathogens (Dorman & Short, 2017). The injudicious use of antibiotics has accelerated the development of antibiotic resistance. Over the years, there has been an increase of cases caused by multidrug-resistant *K. pneumoniae* and the trends of *K. pneumoniae* strains harbouring resistance genes such as extended spectrum β-lactamase (ESBL) and carbapenemase genes have also been observed (Müller-Schulte et al., 2020; Sirot et al., 1988; Nordmann, Cuzon & Naas, 2009). The propensity for colonisation, transmission, and survival under selection pressure of antibiotics could have enhanced their survival rate, thus contributing to the high infection rate in the hospital setting.

The surveillance of the major carbapenemase genes such as *bla*KPC, *bla*NDM, *bla*VIM, *bla*IMP and *bla*OXA has been carried out in many countries (Hamzan et al., 2015; Rimrang et al., 2012; Hoang et al., 2013; Karuniawati, Saharman & Lestari, 2013). The reported dominant carbapenemase gene in Singapore and China were *bla*NDM and *bla*KPC respectively (Teo et al., 2014; Zhang et al., 2017). However, in our study, *bla*OXA-48 and *bla*NDM were the two main carbapenemase genes detected. *bla*OXA-48 and *bla*NDM carried on IncL group plasmid and IncFIIK plasmid have been reported. These plasmids have been associated with low fitness burden, high plasmid stability and high transconjugation frequency (Chaalal et al., 2021). In addition, close genetic association between the *ble*MBL and *bla*NDM-1 genes has been reported. These two genes are controlled by the same promoter, and this will lead to constitutive expression of *bla*NDM and *ble*MBL that encodes a bleomycin resistant protein (BRP). BRF is a functional protein that prevent DNA damage, thus stabilise the plasmid-borne *bla*NDM. Studies have shown that *bla*BRF present in both non-clinical and clinical environment. In clinical environment, the medical use of bleomycin as an anticancer agent, contribute to selective pressure that led to further spread of *bla*NDM in the environment (Dortet, Nordmann & Poirel, 2012). Finally, the dissemination of carbapenemase genes is possibly affected by characteristics of the primary reservoir (which depends on population density, hygiene level, antibiotic selection pressure in that specific area) and population exchange (imported cases from endemic areas) (Nordmann & Poirel, 2014).

Several carbapenem resistance mechanisms could be used among the strains without carbapenemase genes. For this group of strains, the resistance could be attributed by permeability defect (porin loss or mutation) coupled with combination of AmpC expression; or ESBL expression; or non-targeted carbapenemase genes in this study (Crowley, Benedí & Doménech-Sánchez, 2002; Shin et al., 2012; García-Fernández et al., 2010). On the other hand, ertapenem mono resistant non-carbapenemase producing *K. pneumoniae* (EMRNCP-*K.p*) is also believed to acquire ESBL genes and/or alteration of porins (Chung, Yong & Lee, 2016). In our present study, only a small number of strains showed the loss of porins that could possibly changes the outer membrane permeability. These group of strains were believed to survive poorly compared to other strains. Previous studies had shown that carbapenem resistant bacteria without porins are usually unstable, thus conferred lower fitness, growth and transmission ability in the hospital environment (Nordmann, Dortet & Poirel, 2012). However, it is noteworthy that the presence of porin associated genes may not guarantee the expression. Expression of porin can also be disrupted by insertional inactivation that alters translation resulting in early termination of translation (Chung, Yong & Lee, 2016; Kaczmarek et al., 2006).

Majority of the CRKp strains co-harbouring *bla*NDM and *bla*OXA-48 belonged to Cluster I. On the other hand, 1 strain that did not harbour any targeted carbapenemase genes also grouped in Cluster I. Hence, it is suggested that strains that shared the same genetic backbone might not utilising the same resistance mechanisms.

Although carbapenemase plays an important role in carbapenem resistance, but carbapenemase did not affect the mortality of patients with CRE infection based on statistical analysis. This agrees with previous studies (Daikos et al., 2009). However, there are studies reported the correlations between mortality and severity of underlying diseases as well as the degree of carbapenem resistance (based on MIC value) (Daikos et al., 2009; Daikos et al., 2014). In our study, patients who received definitive treatment at more than 3 days from sample isolation date was significantly associated with mortality compared to patients that received definitive treatment at less or equal than 3 days from sample isolation date. The rapid initiation of appropriate therapy (definitive treatment) has previously been shown to affect in-hospital mortality, but highly dependent on rapid diagnostic that produce accurate and reliable results (Deresinski, 2007; Kumar et al., 2006). In addition, previous studies also suggested the importance of active screening of patients colonised with carbapenemase-producing CRE for implementation of infection control measures, such as isolation, precautions during invasive procedures, patient transport and environment disinfection (Banerjee & Humphries, 2017).

Based on the comparison of the finding reported by Low et al. (2017) with our current study, the trend of carbapenem-resistance mechanisms has been changed from year 2013 to 2015. Overall, more resistance mechanisms have been observed in year 2013 compared to 2014 and 2015. For instance, *bla*KPC-2 and *bla*IMP have been observed in 2013 but not detected in the latter years. The previous study found that CRKp strains with *bla*KPC in 2013 were associated with Tn*4401*b transposon (Low et al., 2017). This isoform has been reported to exhibit lower resistance towards carbapenems when compared to other isoforms (Kitchel et al., 2010). Further, these strains did not belong to the high-risk clones that are responsible for the spread of KPC-harbouring *K. pneumoniae* such as ST258 and ST11 (Lee et al., 2016), hence it only appeared sporadically. On the other hand, in 2013, only 1 strain co-harbouring *bla*NDM and *bla*IMP-8 were found but *bla*NDM has been the main resistance mechanisms among the strains in 2014 although superseded by *bla*OXA-48 in 2015. Moreover, the increase of strains that did not harbour the five targeted carbapenemase genes has been observed since 2014. In addition, only *bla*OXA-48 variant was detected among the strains in 2013 and 2014 but in 2015, 3 *bla*OXA-232 variants were observed. To date, only 2 *bla*OXA variants (*bla*OXA-48, *bla*OXA-181) have been reported in Malaysia (Lau et al., 2021; Lau et al., 2020). This study shed some lights on the changes of main carbapenem-resistance mechanisms in the hospital within the 3-year period; however, further study is required with more recent patient data set to track the genetic shift of these clinical bacterial strains.

The in-hospital mortality rate of CRKp in 2013, 2014 and 2015 were 35.3%, 27.3% and 42.6% respectively. When comparing the CRKp strains among the predominant pulsotype, the in-hospital mortality is increasing from 2013 to 2015 in which the mortality rate of 2013, 2014 and 2015 were 11.1%, 16.7% and 69.6% respectively. The reason behind of the increment could not be concluded based on the collected details. A further study which includes host factors such as white blood cells and nutritional status as well as bacterial virulence factors are needed.

## Conclusions

Emergence of CRE was observed in our study setting from 2014 to 2015. The predominant species was *K. pneumoniae*. The genotypic characterisation of CRKp revealed that the predominant pulsotype had circulated in our setting since 2013. The circulation of this pulsotype with more than one carbapenemase gene could have a negative impact on the management of patients. Hence, this study is important to provide better understanding on the spread of CRE and carbapenemase gene.

## Supplemental Information

10.7717/peerj.12830/supp-1 Supplemental Information 1 Dendrogram generated by UPGMA clustering method using Dice coefficient. Dendrogram generated by UPGMA clustering method using Dice coefficient. Among the 140 CRKp strains, seven clusters and 48 pulsotypes were generated.

10.7717/peerj.12830/supp-2 Supplemental Information 2 CRE study raw data.

## Additional Information and Declarations

### Data Availability

The following information was supplied regarding data availability: The raw gel pattern, sample isolation date, sample isolation ward, presence of carbapenemase and porins are presented in dendrograms are available in the Supplemental Files.
