# Supplementary figures and images for: A retrospective study on molecular epidemiology trends of carbapenem resistant Enterobacteriaceae in a teaching hospital in Malaysia
Source: PeerJ. 2022 Feb 22;10:e12830. doi: 10.7717/peerj.12830 (PMC8877335; doi:10.7717/peerj.12830)

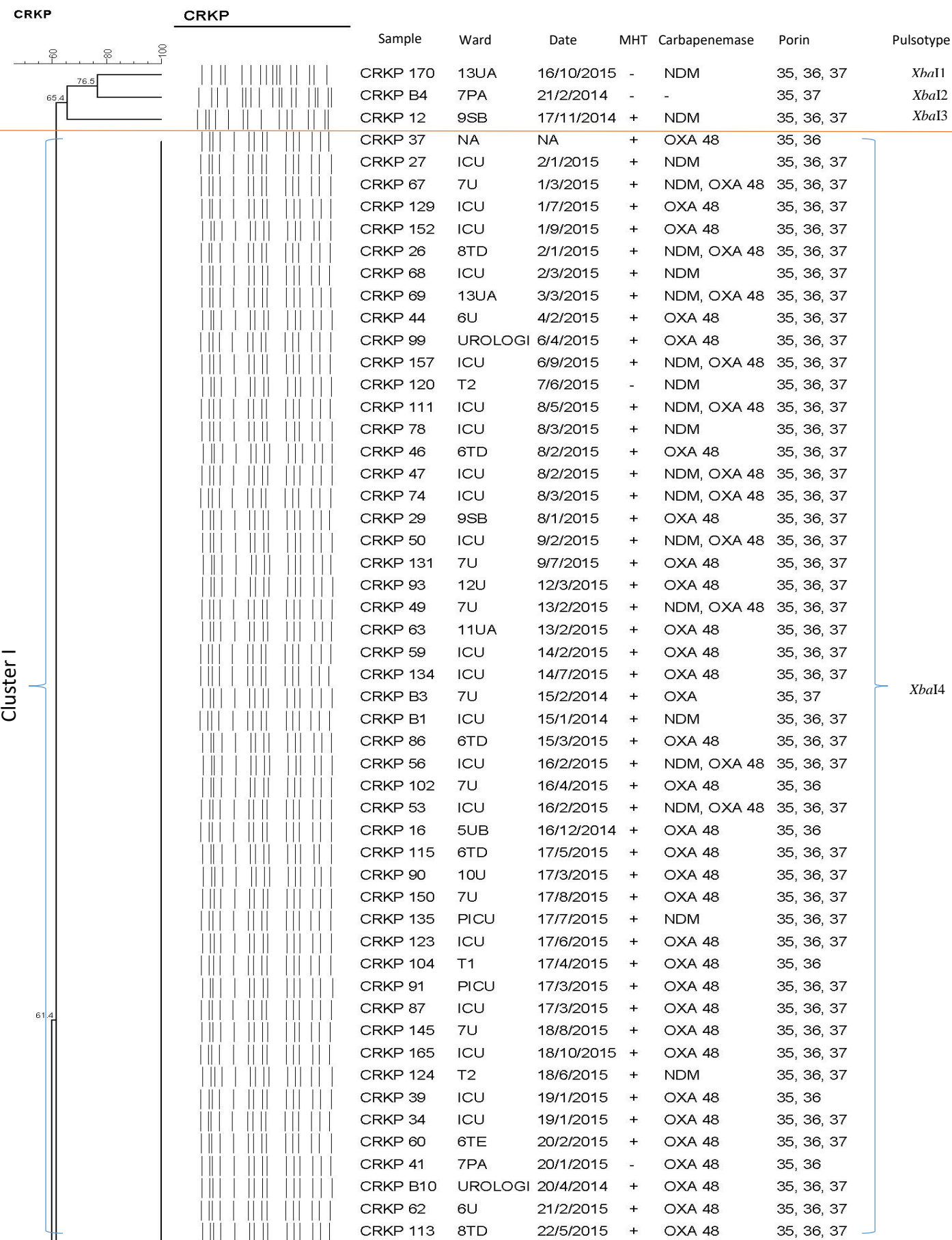

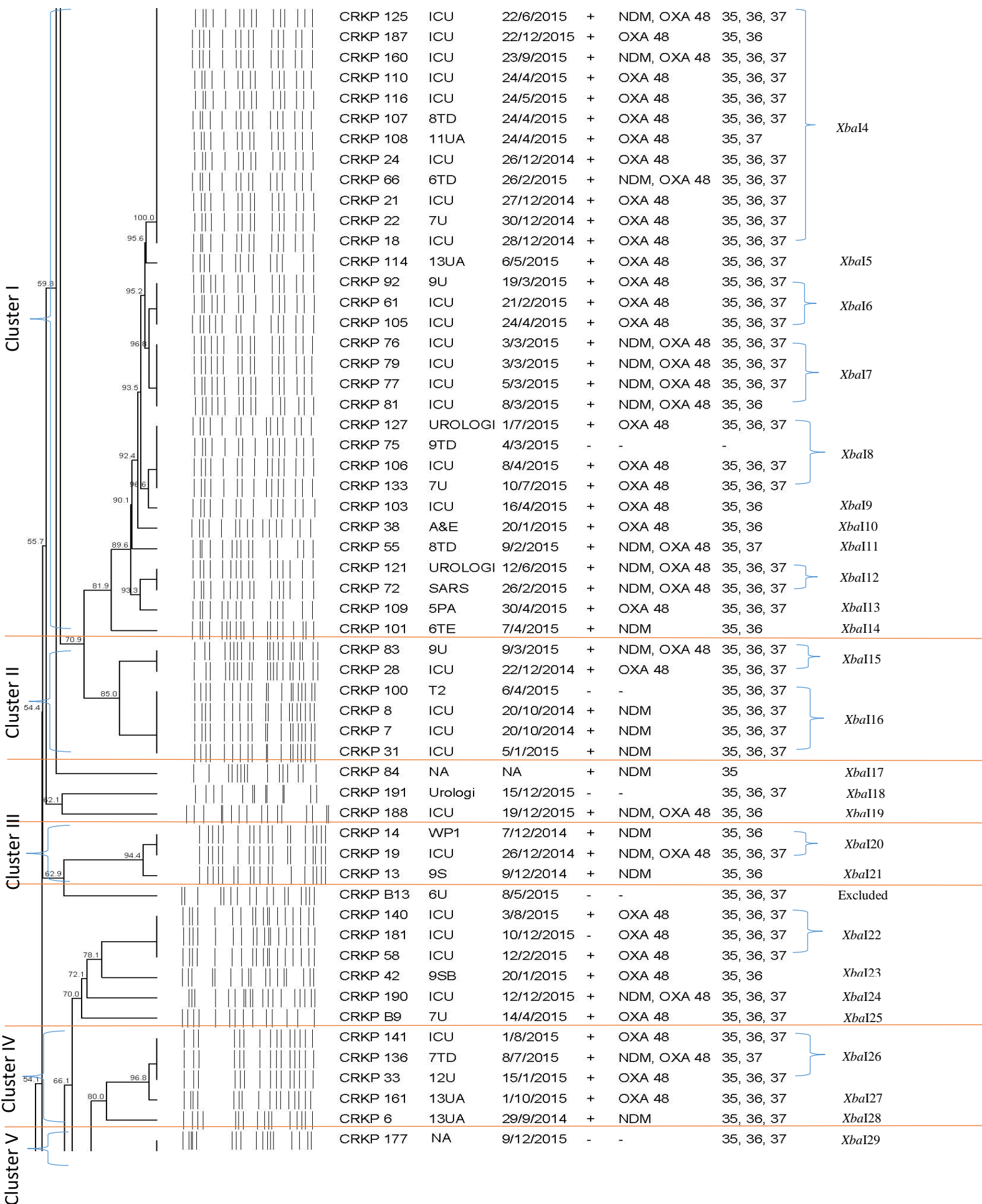

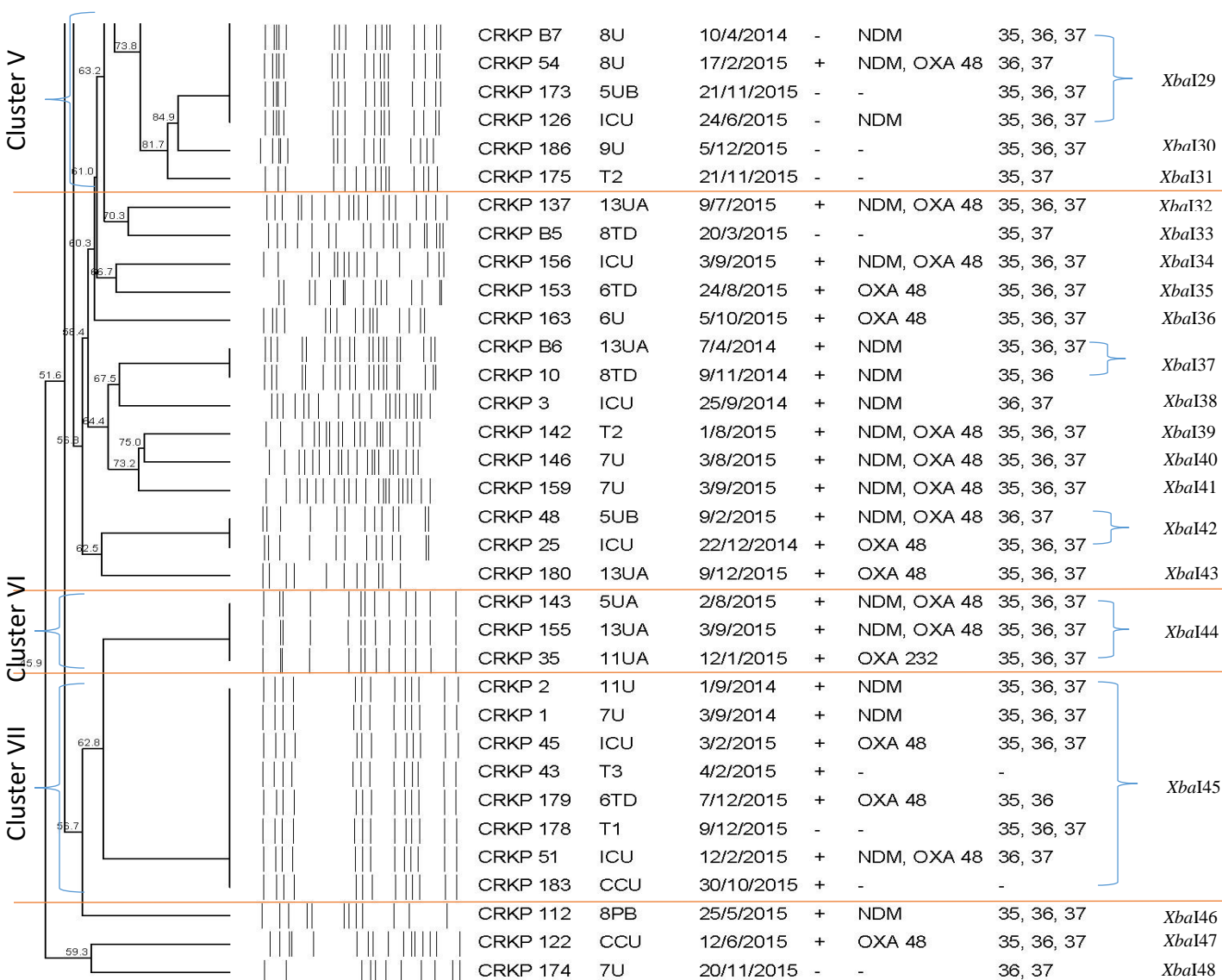

S1-S3: Dendrogram generated by UPGMA clustering method using Dice coefficient.

Supplement: Supplemental Information 1 — Dendrogram generated by UPGMA clustering method using Dice coefficient. Among the 140 CRKp strains, seven clusters and 48 pulsotypes were generated. [file peerj-10-12830-s001.pdf]
